# Supplementary material for: A novel SARS-CoV-2 related coronavirus in bats from Cambodia
Source: Nat Commun. 2021 Nov 9;12:6563. doi: 10.1038/s41467-021-26809-4 (PMC8578604; doi:10.1038/s41467-021-26809-4)
Supplement: Supplementary file 1 — Supplementary Information [file 41467_2021_26809_MOESM1_ESM.pdf]

Supplementary figures and tables for

A novel SARS-CoV-2 related coronavirus in bats from Cambodia

Deborah Delaune[1,2,3,*], Vibol Hul[4,*], , Erik A. Karlsson[4,*], Alexandre Hassanin[5], Tey Putita Ou[4], Artem Baidaliuk[1], Fabiana Gámbaro[1,6], Matthieu Prot[1], Vuong Tan Tu[5,7], Sokha Chea[8], Lucy Keatts[9,10], Jonna Mazet[10], Christine K. Johnson[10], Philippe Buchy[4,11], Philippe Dussart[4,12], Tracey Goldstein[10], Etienne Simon-Lorière[1,$], Veasna Duong[4,$]

[1]Evolutionary Genomics of RNA Viruses, Department of Virology, Institut Pasteur, Paris, France.
[2]Institut de Recherche Biomédicale des Armées, Brétigny-sur-Orge, France.
[3]Université Paris-Saclay, Orsay, France.
[4]Virology Unit, Institut Pasteur du Cambodge, Institut Pasteur International Network, Phnom Penh, Cambodia.
[5]Institut de Systématique, Évolution, Biodiversité, Sorbonne Université, MNHN, CNRS, EPHE, UA, Paris, France.
[6]Université de Paris, Sorbonne Paris Cité, Paris.
[7]present address: Institute of Ecology and Biological Resources, Vietnam Academy of Science and Technology, Hanoi, Vietnam.
[8]Wildlife Conservation Society, Cambodia Program, Phnom Penh, Cambodia
[9]Wildlife Conservation Society, Health Program, Bronx, New York, USA.
[10]One Health Institute, School of Veterinary Medicine, University of California, Davis, USA.
[11]present address: GlaxoSmithKline Vaccines R&D Greater China & Intercontinental, Singapore, Singapore.
[12]present address: Virology Unit, Institut Pasteur de Madagascar, Institut Pasteur International Network, Antananarivo, Madagascar.

*: These authors contributed equally
[$]: These authors jointly supervised this work

Correspondence: etienne.simon-loriere@pasteur.fr or dveasna@pasteur-kh.org

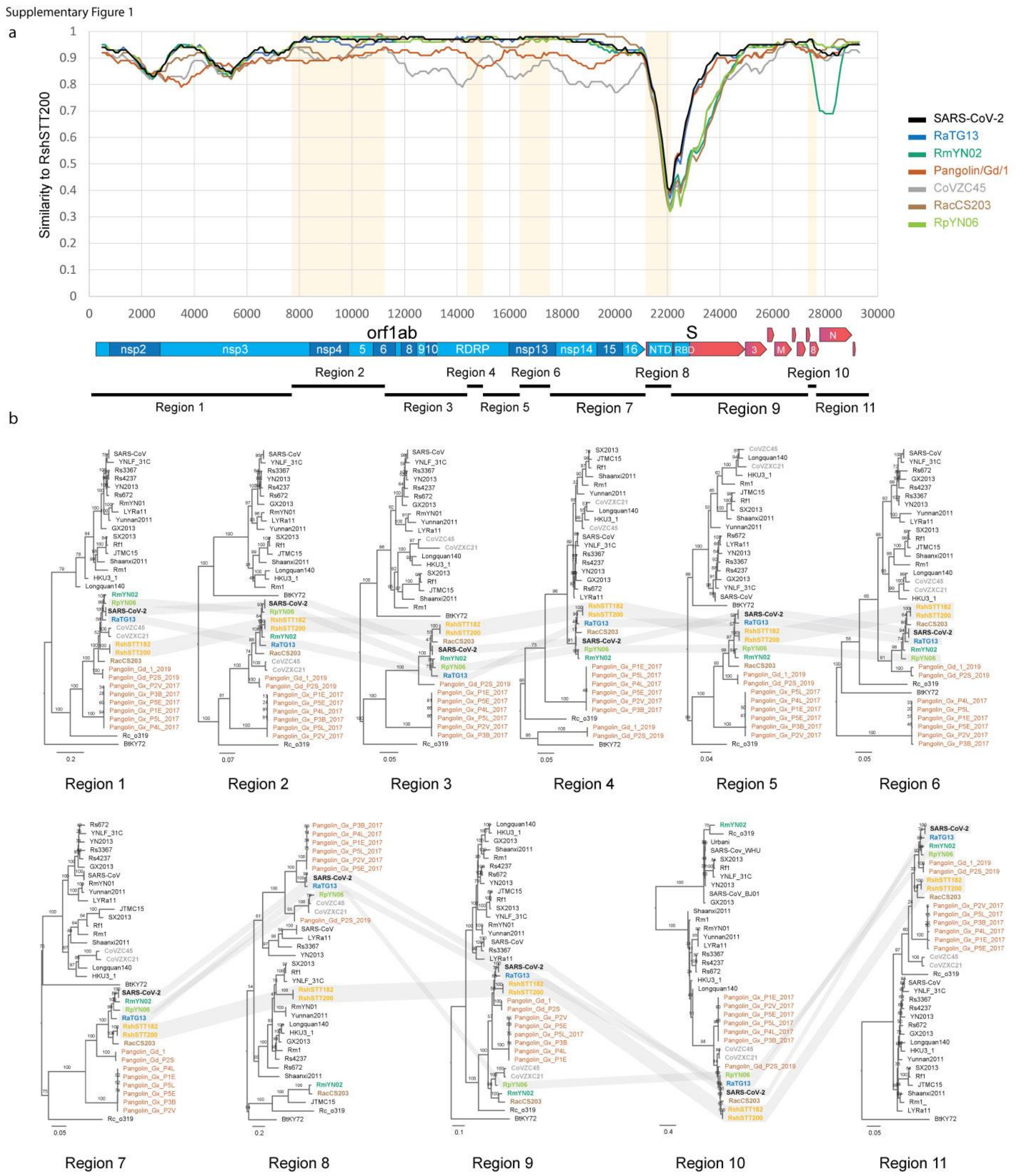

Supplementary figure 1. Recombination analysis. a. Sliding window analysis of changing patterns of sequence similarity between RshSTT200 and related coronaviruses from China and Cambodia. ZXC21 and ZC45 were merged for this analysis. The grey shaded boxes indicate regions of RshSTT182/RshSTT200 genomes identified as recombinant. These potential breakpoints subdivide the genomes into 11 regions, indicated by the black bars at the bottom of the similarity plot. The genome organization with the predicted ORFs is shown. b. Phylogenetic tree of genomic regions defined by the recombination analysis. Branch support obtained from 1,000 bootstrap replicates are indicated. SARS-CoV-2 sequences are collapsed, and trees are midpoint rooted for clarity.

Supplementary Figure 2

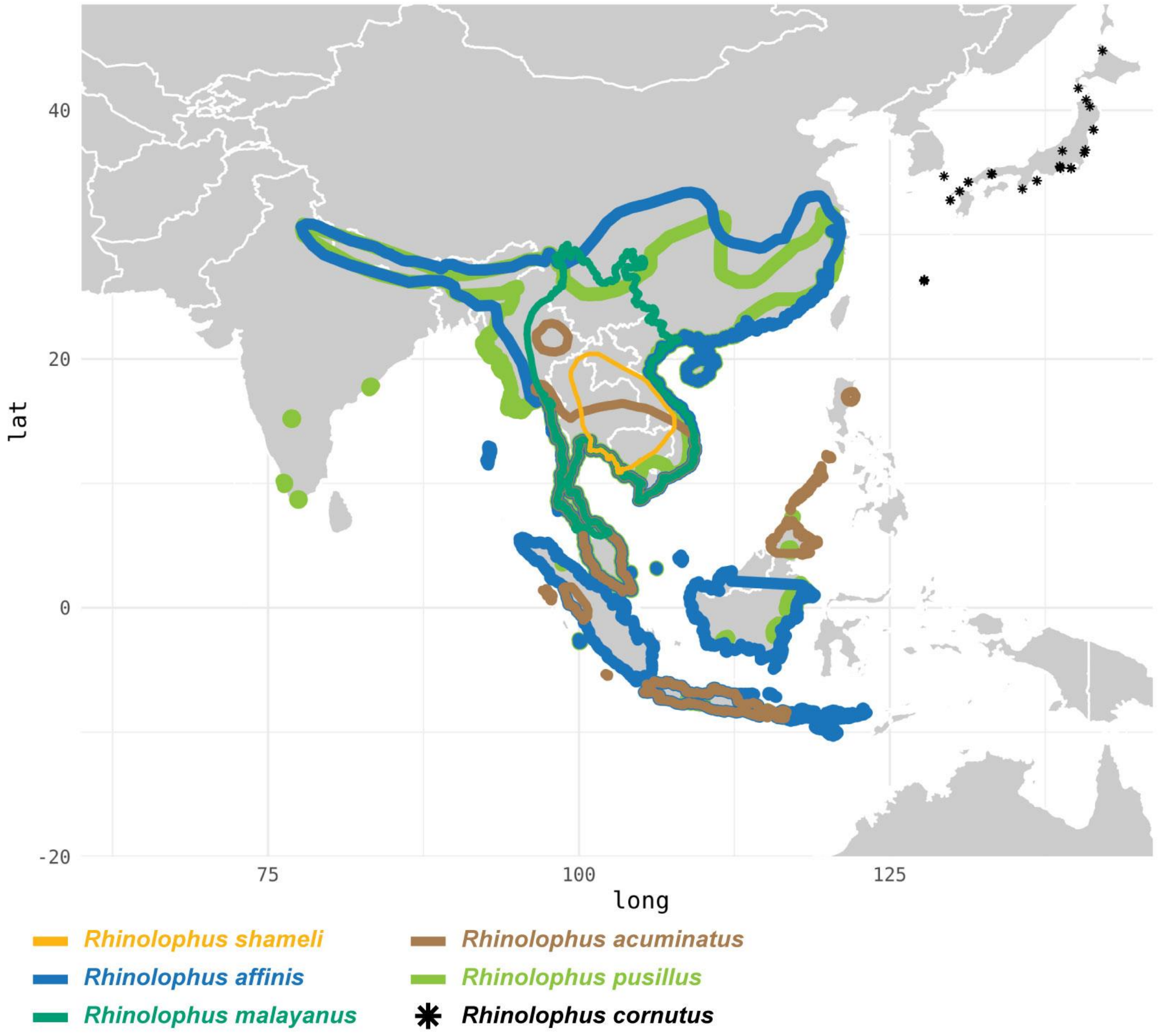

Supplementary figure 2. Bats species distribution in South East Asia related to SARS-CoV-2-like virus detection. The distribution of *R. shameli*[1], *R. acuminatus*[2], *R. affinis*[3], *R. pusillus*[4] *and R. malayanus*[5] are shown in orange, brown, blue, light green, and dark green, respectively. Occurrences of *R. cornutus* are shown with a black star. Data retrieved from The IUCN Red List of Threatened Species (https://www.iucnredlist.org/) and the Global Biodiversity Information Facility (https://www.gbif.org/).

Supplementary Figure 3

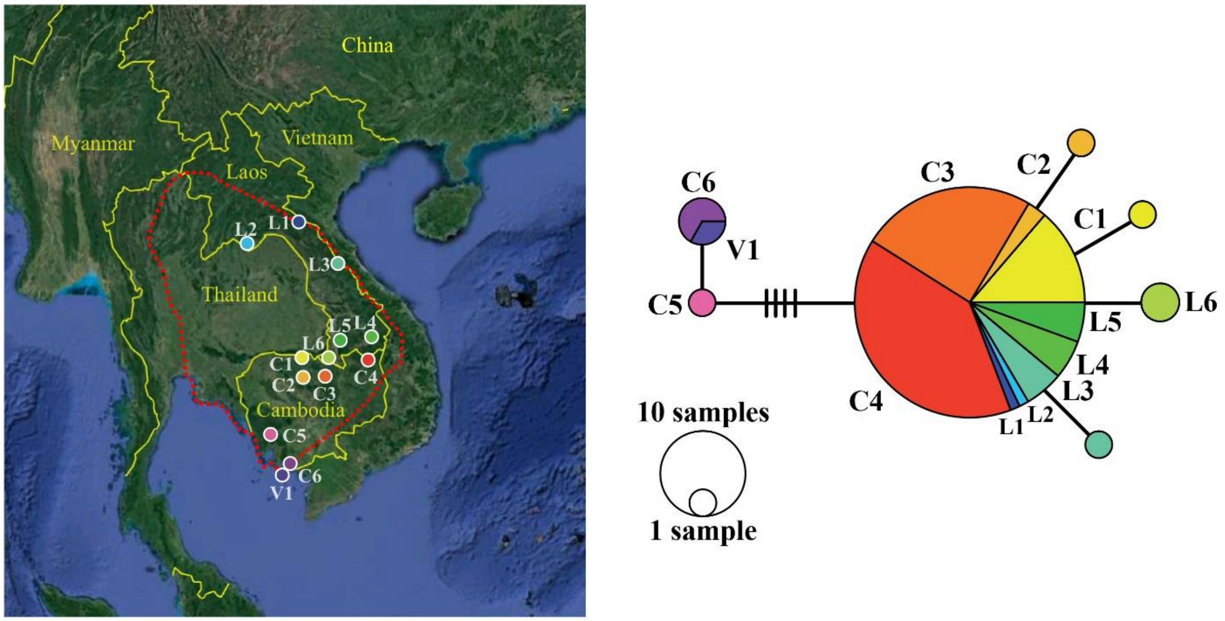

Supplementary figure 3. Haplotype network based on 82 cytochrome c oxidase subunit 1 gene (CO1) sequences of *Rhinolophus shameli* sampled in 13 localities. The network was constructed with the median joining method available in PopART 1.5 (www.popart.otago.ac.nz/) using equal weights for all mutations. The 13 localities where *R. shameli* bats were sampled are shown in the map. They are distributed in three countries (C: Cambodia; L: Laos; V: Vietnam): C1: Preah Vihear Temple; C2: Krong; C3: Sro Lav; C4: Virachey National Park; C5: Kaoh Kong; C6: Kampot; L1: Nam Kading; L2: Vientiane; L3: Hin Nam No; L4: Attapeu; L5: Dong Hua Sao; L6: Dong Kanthung; V1: PhuQuoc. The red dashes indicate the IUCN geographic range of *R. shameli*[1]. Map from Google Earth US Dept of State Geographer © 2020 Google – Image Landsat / Copernicus - Data SIO, NOAA, U.S. Navy, NGA, GEBCO.

Supplementary Figure 4

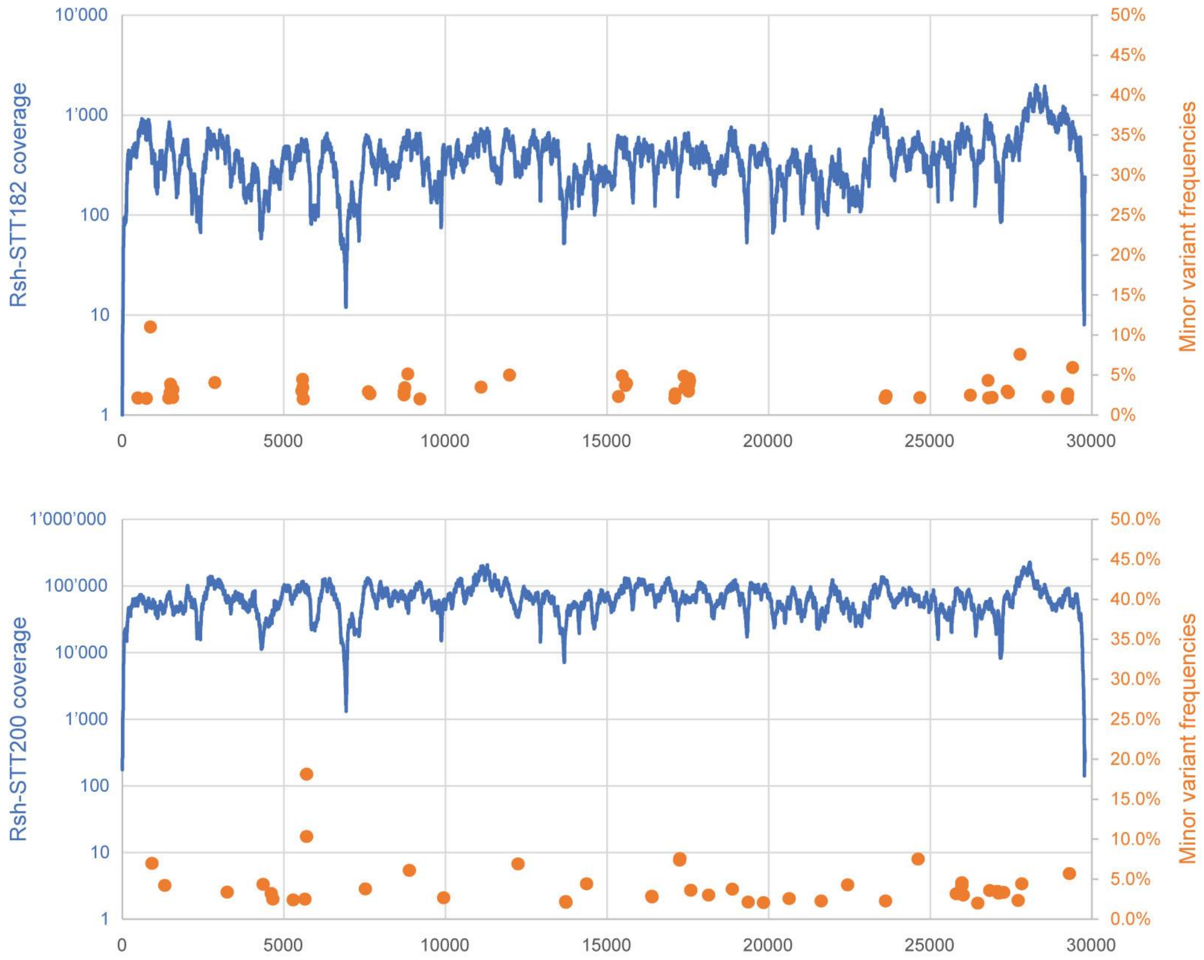

Supplementary figure 4. Reads coverage and frequency of minor variants for both coronaviruses from Cambodia

Supplementary table 1. Distribution of positive samples within the species for retrospective analyzed samples. Sarbecoviruses were only detected in *Rhinolophulus shameli* bat species samples.

Families/species	Number of Sample	Number of positive samples by pan-CoV hemi-nested RT-PCR	Number of positive samples by real time RT-PCR RDRP gene
Emballonuridae			
Taphozous longimanus	10	0	0
Hipposideridae			
Hipposideros galeritus	1	0	0
Hipposideros pomona	2	0	0
Megadermatidae			
Megaderma lyra	2	0	0
Pteropodidae			
Cynopterus brachyotis	14	0	0
Cynopterus sphinx	173	2	0
Cynopterus sp.	2	0	0
Macroglossus minimus	50	0	0
Macroglossus sobrinus	12	0	0
Rhinolophidae			
Rhinolophus affinis	9	0	0
Rhinolophus malayanus	1	0	0
Rhinolophus shameli	35	4	2
Vespertilionidae			
Myotis horsfieldii	4	0	0
Myotis muricola	18	0	0
Pipistrellus javanicus	2	0	0
Scotophilus kuhlii	65	10	0
Tylonycteris robustula	1	0	0
Viverridae			
Paguma larvata	4	0	0
Paradoxurus hermaphroditus	18	0	0
Paradoxurus sp.	1	0	0
Viverra megaspila	2	0	0
Viverra sp.	2	0	0
Felidae			
Prionailurus viverrinus	2	0	0
Total	*430*	*16*	*2*

Supplementary table 2. Sequence identity for SARS-CoV-2 compared with RshSTT182/RshSTT200 and representative BetaCoV genomes.

nucleotides	complete genome	gene													
		orf1ab	RDRP	S	RBD	RBM	NTD	ofr3a	E	M	orf6	orf7	orf8	N	orf10
RshSTT182	92,6	94,5	97,1	79,6	78	63,4	54,1	94,3	99,1	95	95,6	96,7	92	93,8	99,1
RshSTT200	92,6	94,5	97,1	79,6	78	63,4	54,1	94,3	99,1	95	95,6	96,7	92	93,8	99,1
bat/Yunnan/RaTG13/2013	95,9	96,5	97,8	92,8	86,2	90,7	88,7	96,2	99,5	95,5	98,3	96,5	96,9	96,9	99,1
bat/Yunnan/RpYN06/2020	94,2	97,1	98,3	75,9	62,3	61,3	57	96,2	99,1	93,4	96,7	96,9	97,5	97,6	100
bat/Yunnan/RmYN02/2019	92,8	97,1	98,3	71,2	62	50,6	39	96,3	98,6	94,6	96,7	95,1	41,7	97,3	99,1
bat/Thailand/RacCS203/2020	90,8	94,1	96,6	69,6	61,5	50,5	39,5	91,9	99,1	94	96,2	92,7	92	93,1	99,1
bat_SL_CoVZC45	87,4	88,9	86,5	75,2	63,3	63,3	59,6	87,8	98,6	93,4	95,1	90,2	88,5	91,1	99,1
bat_SL_CoVZXC21	87,2	88,6	86,8	74,7	62	62,6	60,4	88,8	98,6	93,4	95,1	90,6	88,5	91,1	/
pangolin/Guangdong/1/2019	90	90,2	91,3	83,6	86,8	73,1	62,7	93,2	99,1	93,2	95,6	92,9	92,3	96,1	99,1
pangolin/Guangxi/P5L/2017	85	84,5	88,5	83	79,8	78,6	72	86,9	97,3	91,3	91,4	85,2	80,6	90,8	94

amino acids	complete genome	gene													
		orf1ab	RDRP	S	RBD	RBM	NTD	ofr3a	E	M	orf6	orf7	orf8	N	orf10
RshSTT182	NA	92,9	91,6	73,8	84,3	62,6	53,4	96	98,6	99	87,5	95,6	91,7	96,6	97,3
RshSTT200	NA	92,9	91,6	73,8	84,3	62,6	53,4	96	98,6	99	87,5	95,6	91,7	96,6	97,3
bat/Yunnan/RaTG13/2013	NA	96	94,1	90,5	90,1	95	96,5	97,8	100	99,5	94,6	97,5	95	99	97,3
bat/Yunnan/RpYN06/2020	NA	96,2	95,5	68	66,8	63,4	56,3	96,7	100	98,1	89,2	96,8	96,6	99,2	100
bat/Yunnan/RmYN02/2019	NA	96,3	95,5	63	64,1	47,9	32,9	96,7	100	98,6	89,2	92,5	28,5	98,5	97,3
bat/Thailand/RacCS203/2020	NA	93	89,9	58,8	64,5	47,2	32,5	97,4	100	98,1	90,9	93,1	94,2	95,7	97,3
bat_SL_CoVZC45	NA	83,6	65	64,8	66,8	64,9	58,1	90,9	100	98,6	87,2	87,5	94,2	94,2	97,3
bat_SL_CoVZXC21	NA	83,5	66	63,8	66,8	63,7	55,8	92	100	98,6	87,2	88,1	94,2	94,2	/
pangolin/Guangdong/1/2019	NA	87,5	75	78,5	96,8	78,4	58,1	97,1	100	98,6	89	93,1	95	97,8	97,3
pangolin/Guangxi/P5L/2017	NA	80,5	68,6	77,4	86,5	87,4	81,3	90,2	100	98,1	75,4	79,5	87,6	93,7	84,2

NA: not available, (/): the open reading frame is not found

Sequence identities for SARS-CoV-2 consensus (Genbank accession number NC_045512.2) compared with the RshSTT182 & RshSTT200 (EPI_ISL_852604 and EPI_ISL_852605); the bat SARS-like coronaviruses RaTG13 (EPI_ISL_402131), RpYN06 (MZ081381), RmYN02 (EPI_ISL_412977), RacCS203 (MW251308), ZC45 (MG772933), ZXC21 (MG772934); the pangolin SARS-like coronaviruses pangolin/GD/2019 (EPI_ISL_410721) and pangolin/GX/P5L/2017 (EPI_ISL_410540).

Supplementary table 3. a. Viral scaffolds identified in this study for sample STT200.

sample_id	host_species	scaffold_id	scaffold_length_nt	scaffold_kmer_coverage	hit_accession	hit_pident_aa	hit_length_aa	hit_bitscore	hit_superkingdom	hit_realm	hit_kingdom	hit_phylum	hit_class	hit_order	hit_family	hit_genus	hit_species
STT200	Rhinolophus shameli	594	2138	5.983197	YP_009553584.1	32.8	591	256.9	Viruses								Elicom_virus_1
STT200	Rhinolophus shameli	1932	863	24.289604	AMO03225.1	35.3	241	125.2	Viruses								Marsac_virus
STT200	Rhinolophus shameli	8099	313	6.209302	AKH66967.1	35.4	99	74.7	Viruses								Motts_Mill_virus
STT200	Rhinolophus shameli	14173	220	7.569697	AKH40292.1	34.3	67	55.5	Viruses								Motts_Mill_virus
STT200	Rhinolophus shameli	6259	376	2.950156	ASF00830.1	100	124	261.2	Viruses								uncultured_virus
STT200	Rhinolophus shameli	9676	280	2.373333	ASF00828.1	100	93	189.9	Viruses								uncultured_virus
STT200	Rhinolophus shameli	702	1849	377.54738	APG76171.1	31	477	165.6	Viruses	Riboviria							Beihai_noda-like_virus_17
STT200	Rhinolophus shameli	12252	242	1.796791	AYP67543.1	46.6	73	73.2	Viruses	Riboviria							Blue_fish_point_virus
STT200	Rhinolophus shameli	3389	582	7.73055	BBQ04782.1	42.1	107	71.6	Viruses	Riboviria							Culex_inatomii_luteo-like_virus
STT200	Rhinolophus shameli	12769	235	4.738889	YP_009337365.1	39.4	66	55.5	Viruses	Riboviria							Hubei_permutotetra-like_virus_7
STT200	Rhinolophus shameli	365	4621	166.029566	YP_009337778.1	50.1	1032	1003.4	Viruses	Riboviria							Hubei_permutotetra-like_virus_9
STT200	Rhinolophus shameli	15444	209	14.564935	YP_009337778.1	59.7	62	72	Viruses	Riboviria							Hubei_permutotetra-like_virus_9
STT200	Rhinolophus shameli	1686	946	4.842873	YP_009337003.1	49.5	313	335.9	Viruses	Riboviria							Hubei_picorna-like_virus_26
STT200	Rhinolophus shameli	4781	455	3.51	YP_009337003.1	44.2	154	121.7	Viruses	Riboviria							Hubei_picorna-like_virus_26
STT200	Rhinolophus shameli	9553	282	2.572687	YP_009337003.1	55.2	87	110.5	Viruses	Riboviria							Hubei_picorna-like_virus_26
STT200	Rhinolophus shameli	14134	221	2.554217	YP_009337118.1	52.4	63	68.6	Viruses	Riboviria							Hubei_picorna-like_virus_52
STT200	Rhinolophus shameli	475	2770	188.703499	YP_009330010.1	63.1	369	505	Viruses	Riboviria							Hubei_sobemo-like_virus_10
STT200	Rhinolophus shameli	504	2582	7.284923	YP_009329960.1	56.4	415	487.3	Viruses	Riboviria							Hubei_sobemo-like_virus_43
STT200	Rhinolophus shameli	11635	250	2.246154	YP_009330002.1	58.5	82	99	Viruses	Riboviria							Hubei_sobemo-like_virus_46
STT200	Rhinolophus shameli	919	1522	12.686435	APG75767.1	40	170	122.5	Viruses	Riboviria							Hubei_sobemo-like_virus_48
STT200	Rhinolophus shameli	255	8989	76.410343	AVK59469.1	30.7	2276	924.5	Viruses	Riboviria							Hubei_virga-like_virus_11
STT200	Rhinolophus shameli	4117	506	4.177384	QFR59037.1	41.8	158	106.7	Viruses	Riboviria							Jiangan_virus
STT200	Rhinolophus shameli	11777	248	2.145078	QFR59037.1	49	49	53.1	Viruses	Riboviria							Jiangan_virus
STT200	Rhinolophus shameli	14282	219	6.310976	YP_009333214.1	71.4	70	102.4	Viruses	Riboviria							narna-like_virus_6
STT200	Rhinolophus shameli	15572	208	77.130719	QKN89003.1	49.3	67	61.6	Viruses	Riboviria							Riboviria_sp.
STT200	Rhinolophus shameli	489	2670	28.966348	APG75881.1	55.4	341	407.9	Viruses	Riboviria							Sanxia_water_strider_virus_12
STT200	Rhinolophus shameli	1348	1123	3.745318	YP_009337272.1	41.9	375	255.8	Viruses	Riboviria							Shuangao_arthropod_virus_9
STT200	Rhinolophus shameli	17817	110	11.727273	YP_009337273.1	74.3	35	70.5	Viruses	Riboviria							Shuangao_arthropod_virus_9
STT200	Rhinolophus shameli	8851	296	2.614108	YP_009337273.1	39.8	83	59.3	Viruses	Riboviria							Shuangao_arthropod_virus_9
STT200	Rhinolophus shameli	4014	515	4.152174	YP_009342307.1	49.1	159	166.4	Viruses	Riboviria							Wuhan_arthropod_virus_4
STT200	Rhinolophus shameli	7365	334	4.956989	YP_009342307.1	51.4	111	117.1	Viruses	Riboviria							Wuhan_arthropod_virus_4
STT200	Rhinolophus shameli	3693	547	2.721545	YP_009342306.1	53.8	52	68.2	Viruses	Riboviria							Wuhan_arthropod_virus_4
STT200	Rhinolophus shameli	13932	223	1.410714	YP_009342053.1	44.9	69	68.6	Viruses	Riboviria							Wuhan_coneheads_virus_1
STT200	Rhinolophus shameli	969	1454	201.962831	YP_009342462.1	34.5	252	138.7	Viruses	Riboviria							Wuhan_house_centipede_virus_4
STT200	Rhinolophus shameli	842	1607	522.899485	YP_009342449.1	38.2	225	135.2	Viruses	Riboviria							Wuhan_house_centipede_virus_5
STT200	Rhinolophus shameli	902	1546	451.579477	YP_009342449.1	31.9	160	90.1	Viruses	Riboviria							Wuhan_house_centipede_virus_5
STT200	Rhinolophus shameli	1083	1347	175.842879	AWV67033.1	35.3	400	237.7	Viruses	Riboviria	Orthornavirae	Duplornaviricota	Resentoviricetes	Reovirales	Reoviridae	Rotavirus	Rotavirus_H
STT200	Rhinolophus shameli	414	3535	299.156609	APQ41753.1	67.5	1163	1526.5	Viruses	Riboviria	Orthornavirae	Duplornaviricota	Resentoviricetes	Reovirales	Reoviridae	Rotavirus	Rotavirus_J
STT200	Rhinolophus shameli	445	3074	332.881749	APQ41754.1	65.7	997	1308.9	Viruses	Riboviria	Orthornavirae	Duplornaviricota	Resentoviricetes	Reovirales	Reoviridae	Rotavirus	Rotavirus_J
STT200	Rhinolophus shameli	600	2122	4202.259313	APQ41755.1	54.6	700	795.4	Viruses	Riboviria	Orthornavirae	Duplornaviricota	Resentoviricetes	Reovirales	Reoviridae	Rotavirus	Rotavirus_J
STT200	Rhinolophus shameli	510	2540	155.603219	APQ41756.1	40.8	835	585.1	Viruses	Riboviria	Orthornavirae	Duplornaviricota	ResentoviricetesU42	Reovirales	Reoviridae	Rotavirus	Rotavirus_J
STT200	Rhinolophus shameli	1119	1306	26.123102	APQ41757.1	72.9	395	581.6	Viruses	Riboviria	Orthornavirae	Duplornaviricota	Resentoviricetes	Reovirales	Reoviridae	Rotavirus	Rotavirus_J
STT200	Rhinolophus shameli	1613	978	55.767064	APQ41748.1	67.1	298	412.9	Viruses	Riboviria	Orthornavirae	Duplornaviricota	Resentoviricetes	Reovirales	Reoviridae	Rotavirus	Rotavirus_J
STT200	Rhinolophus shameli	1413	1077	65.763209	APQ41749.1	46.6	251	245.7	Viruses	Riboviria	Orthornavirae	Duplornaviricota	Resentoviricetes	Reovirales	Reoviridae	Rotavirus	Rotavirus_J
STT200	Rhinolophus shameli	2253	774	11411.731572	APQ41759.1	44.6	240	209.5	Viruses	Riboviria	Orthornavirae	Duplornaviricota	Resentoviricetes	Reovirales	Reoviridae	Rotavirus	Rotavirus_J
STT200	Rhinolophus shameli	2689	683	127.707006	APQ41759.1	42.3	215	182.2	Viruses	Riboviria	Orthornavirae	Duplornaviricota	Resentoviricetes	Reovirales	Reoviridae	Rotavirus	Rotavirus_J
STT200	Rhinolophus shameli	2330	752	49.969871	APQ41751.1	35.8	193	105.9	Viruses	Riboviria	Orthornavirae	Duplornaviricota	Resentoviricetes	Reovirales	Reoviridae	Rotavirus	Rotavirus_J
STT200	Rhinolophus shameli	2237	779	75.281768	APQ41750.1	32.9	164	102.8	Viruses	Riboviria	Orthornavirae	Duplornaviricota	Resentoviricetes	Reovirales	Reoviridae	Rotavirus	Rotavirus_J
STT200	Rhinolophus shameli	308	6358	16.3043	QJI53785.1	42.4	1923	1351.3	Viruses	Riboviria	Orthornavirae	Kitrinoviricota	Alsuviricetes	Hepelivirales	Hepeviridae		Hepeviridae_sp.
STT200	Rhinolophus shameli	576	2191	5.149813	QJI53774.1	30	703	315.8	Viruses	Riboviria	Orthornavirae	Kitrinoviricota	Alsuviricetes	Hepelivirales	Hepeviridae		Hepeviridae_sp.
STT200	Rhinolophus shameli	2774	670	9.58374	AAM77034.1	97.1	137	261.9	Viruses	Riboviria	Orthornavirae	Kitrinoviricota	Flasuviricetes	Amarillovirales	Flaviviridae	Pestivirus	Pestivirus_A
STT200	Rhinolophus shameli	9709	279	4.044643	BAW33238.1	98.9	89	191.8	Viruses	Riboviria	Orthornavirae	Kitrinoviricota	Flasuviricetes	Amarillovirales	Flaviviridae	Pestivirus	Pestivirus_A
STT200	Rhinolophus shameli	9462	283	253.192982	AAD44042.1	100	93	182.6	Viruses	Riboviria	Orthornavirae	Kitrinoviricota	Flasuviricetes	Amarillovirales	Flaviviridae	Pestivirus	Pestivirus_B
STT200	Rhinolophus shameli	949	1476	21.496833	AGZ84317.1	59	446	513.8	Viruses	Riboviria	Orthornavirae	Pisuviricota	Duplopiviricetes	Durnavirales	Partitiviridae		Botryosphaeria_dothidea_partitivirus_1
STT200	Rhinolophus shameli	1384	1099	7.264368	AZT88584.1	73.2	365	559.3	Viruses	Riboviria	Orthornavirae	Pisuviricota	Duplopiviricetes	Durnavirales	Partitiviridae		Delitschia_confertaspora_partitivirus_1
STT200	Rhinolophus shameli	12159	243	2.074468	BCD56385.1	71.3	80	125.9	Viruses	Riboviria	Orthornavirae	Pisuviricota	Duplopiviricetes	Durnavirales	Partitiviridae		Lichen_partiti-like_RNA_virus_2
STT200	Rhinolophus shameli	11942	246	1.848168	BCD56385.1	64.2	81	116.7	Viruses	Riboviria	Orthornavirae	Pisuviricota	Duplopiviricetes	Durnavirales	Partitiviridae		Lichen_partiti-like_RNA_virus_2
STT200	Rhinolophus shameli	14639	216	3.950311	BCD56385.1	50.7	71	80.5	Viruses	Riboviria	Orthornavirae	Pisuviricota	Duplopiviricetes	Durnavirales	Partitiviridae		Lichen_partiti-like_RNA_virus_2
STT200	Rhinolophus shameli	6121	381	5.766871	AZT88608.1	66.1	121	174.9	Viruses	Riboviria	Orthornavirae	Pisuviricota	Duplopiviricetes	Durnavirales	Partitiviridae		Penicillium_brasilianum_partitivirus_1
STT200	Rhinolophus shameli	1078	1351	9.75	QIJ70086.1	65	448	599	Viruses	Riboviria	Orthornavirae	Pisuviricota	Duplopiviricetes	Durnavirales	Partitiviridae		Vandelay_partiti-like_virus
STT200	Rhinolophus shameli	110	24237	16332.170127	QID98793.1	96.9	5322	10326	Viruses	Riboviria	Orthornavirae	Pisuviricota	Pisoniviricetes	Nidovirales	Coronaviridae	Betacoronavirus	Severe_acute_respiratory_syndrome-related_coronavirus
STT200	Rhinolophus shameli	329	5623	14352.221085	QIZ14015.1	92.5	1802	3302.3	Viruses	Riboviria	Orthornavirae	Pisuviricota	Pisoniviricetes	Nidovirales	Coronaviridae	Betacoronavirus	Severe_acute_respiratory_syndrome-related_coronavirus
STT200	Rhinolophus shameli	19042	101	9.630435	QOE78502.1	83.9	31	64.7	Viruses	Riboviria	Orthornavirae	Pisuviricota	Pisoniviricetes	Nidovirales	Coronaviridae	Betacoronavirus	Severe_acute_respiratory_syndrome-related_coronavirus
STT200	Rhinolophus shameli	18828	103	2.833333	QOS50726.1	78.8	33	60.1	Viruses	Riboviria	Orthornavirae	Pisuviricota	Pisoniviricetes	Nidovirales	Coronaviridae	Betacoronavirus	Severe_acute_respiratory_syndrome-related_coronavirus
STT200	Rhinolophus shameli	19765	96	4.95122	QOC61951.1	83.9	31	59.7	Viruses	Riboviria	Orthornavirae	Pisuviricota	Pisoniviricetes	Nidovirales	Coronaviridae	Betacoronavirus	Severe_acute_respiratory_syndrome-related_coronavirus
STT200	Rhinolophus shameli	19673	97	1.642857	QKU32296.1	83.9	31	58.2	Viruses	Riboviria	Orthornavirae	Pisuviricota	Pisoniviricetes	Nidovirales	Coronaviridae	Betacoronavirus	Severe_acute_respiratory_syndrome-related_coronavirus
STT200	Rhinolophus shameli	19698	96	17.585366	QOS50726.1	83.3	30	58.2	Viruses	Riboviria	Orthornavirae	Pisuviricota	Pisoniviricetes	Nidovirales	Coronaviridae	Betacoronavirus	Severe_acute_respiratory_syndrome-related_coronavirus
STT200	Rhinolophus shameli	18147	108	7.735849	QOE78502.1	80.6	31	57.8	Viruses	Riboviria	Orthornavirae	Pisuviricota	Pisoniviricetes	Nidovirales	Coronaviridae	Betacoronavirus	Severe_acute_respiratory_syndrome-related_coronavirus
STT200	Rhinolophus shameli	19062	101	7.195652	ATO98218.1	77.4	31	55.1	Viruses	Riboviria	Orthornavirae	Pisuviricota	Pisoniviricetes	Nidovirales	Coronaviridae	Betacoronavirus	Severe_acute_respiratory_syndrome-related_coronavirus
STT200	Rhinolophus shameli	18166	108	4.603774	QOC61951.1	67.6	34	52.8	Viruses	Riboviria	Orthornavirae	Pisuviricota	Pisoniviricetes	Nidovirales	Coronaviridae	Betacoronavirus	Severe_acute_respiratory_syndrome-related_coronavirus
STT200	Rhinolophus shameli	18176	108	3.54717	QOC61951.1	70.6	34	52.8	Viruses	Riboviria	Orthornavirae	Pisuviricota	Pisoniviricetes	Nidovirales	Coronaviridae	Betacoronavirus	Severe_acute_respiratory_syndrome-related_coronavirus
STT200	Rhinolophus shameli	19167	100	8.444444	QMT91156.1	78.6	28	52.8	Viruses	Riboviria	Orthornavirae	Pisuviricota	Pisoniviricetes	Nidovirales	Coronaviridae	Betacoronavirus	Severe_acute_respiratory_syndrome-related_coronavirus
STT200	Rhinolophus shameli	449	3030	6.94084	QIC52838.1	33.8	536	307.4	Viruses	Riboviria	Orthornavirae	Pisuviricota	Pisoniviricetes	Picornavirales			Apple_picorna-like_virus_1
STT200	Rhinolophus shameli	1641	968	4.933187	QKN88956.1	51.2	283	286.6	Viruses	Riboviria	Orthornavirae	Pisuviricota	Pisoniviricetes	Picornavirales			Picornavirales_sp.
STT200	Rhinolophus shameli	4411	482	4.754098	QKN88956.1	45	160	124.8	Viruses	Riboviria	Orthornavirae	Pisuviricota	Pisoniviricetes	Picornavirales			Picornavirales_sp.
STT200	Rhinolophus shameli	13277	230	1.76	AWK77865.1	39.5	76	62	Viruses	Riboviria	Orthornavirae	Pisuviricota	Pisoniviricetes	Picornavirales			Renmark_bee_virus_2
STT200	Rhinolophus shameli	4384	484	2.955711	QNS17457.1	53.8	160	182.6	Viruses	Riboviria	Orthornavirae	Pisuviricota	Pisoniviricetes	Picornavirales			Serbia_picorna-like_virus_2
STT200	Rhinolophus shameli	6726	356	2.853821	QNS17457.1	58.8	119	153.3	Viruses	Riboviria	Orthornavirae	Pisuviricota	Pisoniviricetes	Picornavirales			Serbia_picorna-like_virus_2
STT200	Rhinolophus shameli	9820	277	3.684685	QNS17457.1	54.3	92	110.2	Viruses	Riboviria	Orthornavirae	Pisuviricota	Pisoniviricetes	Picornavirales			Serbia_picorna-like_virus_2
STT200	Rhinolophus shameli	14519	217	3.981481	QNS17457.1	48.6	72	73.2	Viruses	Riboviria	Orthornavirae	Pisuviricota	Pisoniviricetes	Picornavirales			Serbia_picorna-like_virus_2
STT200	Rhinolophus shameli	14342	219	2.469512	QKN89065.1	52.1	71	72.8	Viruses	Riboviria	Orthornavirae	Pisuviricota	Pisoniviricetes	Picornavirales	Iflaviridae		Iflaviridae_sp.
STT200	Rhinolophus shameli	14279	219	7.47561	QBP37020.1	60	70	88.2	Viruses	Riboviria	Orthornavirae	Pisuviricota	Pisoniviricetes	Picornavirales	Iflaviridae		Lampyris_noctiluca_iflavirus_2
STT200	Rhinolophus shameli	7234	338	4.077739	QIJ70025.1	45.9	111	97.1	Viruses	Riboviria	Orthornavirae	Pisuviricota	Pisoniviricetes	Picornavirales	Iflaviridae	Iflavirus	ACT_flea_iflavirus
STT200	Rhinolophus shameli	12024	245	1.705263	YP_009002581.1	74.4	82	123.6	Viruses	Riboviria	Orthornavirae	Pisuviricota	Pisoniviricetes	Picornavirales	Iflaviridae	Iflavirus	Antheraea_pernyi_iflavirus
STT200	Rhinolophus shameli	8034	315	3.176923	QOW95919.1	51.9	104	105.9	Viruses	Riboviria	Orthornavirae	Pisuviricota	Pisoniviricetes	Picornavirales	Iflaviridae	Iflavirus	Aulacophora_lewisii_iflavirus_1
STT200	Rhinolophus shameli	13673	225	5.605882	QOW95919.1	58.1	74	97.8	Viruses	Riboviria	Orthornavirae	Pisuviricota	Pisoniviricetes	Picornavirales	Iflaviridae	Iflavirus	Aulacophora_lewisii_iflavirus_1
STT200	Rhinolophus shameli	14301	219	4.329268	QOW95919.1	55.2	58	65.9	Viruses	Riboviria	Orthornavirae	Pisuviricota	Pisoniviricetes	Picornavirales	Iflaviridae	Iflavirus	Aulacophora_lewisii_iflavirus_1
STT200	Rhinolophus shameli	13812	224	2.786982	QED21508.1	59.6	52	68.9	Viruses	Riboviria	Orthornavirae	Pisuviricota	Pisoniviricetes	Picornavirales	Picornaviridae		Watson_virus
STT200	Rhinolophus shameli	6988	346	4.429553	QED21508.1	39.1	115	67	Viruses	Riboviria	Orthornavirae	Pisuviricota	Pisoniviricetes	Picornavirales	Picornaviridae		Watson_virus
STT200	Rhinolophus shameli	367	4548	44.621189	AVH76464.1	95.4	1428	2772.7	Viruses	Riboviria	Orthornavirae	Pisuviricota	Pisoniviricetes	Picornavirales	Picornaviridae	Kobuvirus	Kobuvirus_sp.
STT200	Rhinolophus shameli	407	3650	36.821419	YP_004782207.1	79.9	1006	1671	Viruses	Riboviria	Orthornavirae	Pisuviricota	Pisoniviricetes	Picornavirales	Picornaviridae	Kobuvirus	Mouse_kobuvirus_M-5/USA/2010
STT200	Rhinolophus shameli	227	11276	151.840567	AXA52568.1	37.6	2259	1386.7	Viruses	Riboviria	Orthornavirae	Pisuviricota	Pisoniviricetes	Picornavirales	Polycipiviridae		Linepithema_humile_polycipivirus_1
STT200	Rhinolophus shameli	623	2021	5.630722	QKE56317.1	28.2	563	224.6	Viruses	Riboviria	Orthornavirae	Pisuviricota	Pisoniviricetes	Picornavirales	Polycipiviridae		Polycipiviridae_sp.
STT200	Rhinolophus shameli	1678	950	5.877095	QKE56317.1	20.9	297	56.2	Viruses	Riboviria	Orthornavirae	Pisuviricota	Pisoniviricetes	Picornavirales	Polycipiviridae		Polycipiviridae_sp.
STT200	Rhinolophus shameli	411	3576	6.111048	YP_009407948.1	31.4	239	104.8	Viruses	Riboviria	Orthornavirae	Pisuviricota	Pisoniviricetes	Picornavirales	Polycipiviridae	Sopolycivirus	Myrmica_scabrinodis_virus_1
STT200	Rhinolophus shameli	1709	935	8.730682	QKI29238.1	42.9	289	212.6	Viruses	Riboviria	Orthornavirae	Pisuviricota	Pisoniviricetes	Sobelivirales	Solemoviridae		Amygdalus_persica_sobemo-like_virus
STT200	Rhinolophus shameli	12098	244	1.57672	QKI29238.1	53	66	72	Viruses	Riboviria	Orthornavirae	Pisuviricota	Pisoniviricetes	Sobelivirales	Solemoviridae		Amygdalus_persica_sobemo-like_virus
STT200	Rhinolophus shameli	10810	261	3.213592	QKI29238.1	40.5	84	65.5	Viruses	Riboviria	Orthornavirae	Pisuviricota	Pisoniviricetes	Sobelivirales	Solemoviridae		Amygdalus_persica_sobemo-like_virus
STT200	Rhinolophus shameli	474	2790	657.160146	QOI91429.1	41.3	409	309.3	Viruses	Riboviria	Orthornavirae	Pisuviricota	Pisoniviricetes	Sobelivirales	Solemoviridae		Guangzhou_sobemo-like_virus
STT200	Rhinolophus shameli	10134	272	3.281106	QHD64767.1	54.3	92	103.6	Viruses	Riboviria	Orthornavirae	Pisuviricota	Pisoniviricetes	Sobelivirales	Solemoviridae		Plasmopara_viticola_lesion_associated_sobemo-like_1
STT200	Rhinolophus shameli	460	2962	575.791882	QIJ70116.1	39	382	270	Viruses	Riboviria	Orthornavirae	Pisuviricota	Pisoniviricetes	Sobelivirales	Solemoviridae		Rosadiaz_solemo-like_virus
STT200	Rhinolophus shameli	8873	295	5.5125	QJI53805.1	61.2	98	147.1	Viruses	Riboviria	Orthornavirae	Pisuviricota	Pisoniviricetes	Sobelivirales	Solemoviridae		Solemoviridae_sp.
STT200	Rhinolophus shameli	3770	539	6.181818	QJI53807.1	51.9	133	140.2	Viruses	Riboviria	Orthornavirae	Pisuviricota	Pisoniviricetes	Sobelivirales	Solemoviridae		Solemoviridae_sp.
STT200	Rhinolophus shameli	1098	1336	5.559719	YP_006905858.1	35	220	116.7	Viruses	Riboviria	Orthornavirae	Pisuviricota	Stelpaviricetes	Stellavirales	Astroviridae		Astrovirus_VA3
STT200	Rhinolophus shameli	8691	299	2.516393	ADI48255.1	73.9	88	136.7	Viruses	Riboviria	Orthornavirae	Pisuviricota	Stelpaviricetes	Stellavirales	Astroviridae		Bat_astrovirus_GF-7a
STT200	Rhinolophus shameli	7318	336	1.914591	ADI48255.1	63.9	72	104.4	Viruses	Riboviria	Orthornavirae	Pisuviricota	Stelpaviricetes	Stellavirales	Astroviridae		Bat_astrovirus_GF-7a
STT200	Rhinolophus shameli	3485	571	5.187984	ACF75854.1	80.6	103	184.5	Viruses	Riboviria	Orthornavirae	Pisuviricota	Stelpaviricetes	Stellavirales	Astroviridae	Mamastrovirus	Bat_astrovirus_1
STT200	Rhinolophus shameli	14388	218	5.981595	ACF75855.1	43.3	67	64.3	Viruses	Riboviria	Orthornavirae	Pisuviricota	Stelpaviricetes	Stellavirales	Astroviridae	Mamastrovirus	Bat_astrovirus_1
STT200	Rhinolophus shameli	363	4635	9.221179	ACN88713.1	71.2	532	828.6	Viruses	Riboviria	Orthornavirae	Pisuviricota	Stelpaviricetes	Stellavirales	Astroviridae	Mamastrovirus	Bat_astrovirus_Ha/Guangxi/LS11/2007
STT200	Rhinolophus shameli	3915	525	2.778723	ACN88713.1	82.1	168	297	Viruses	Riboviria	Orthornavirae	Pisuviricota	Stelpaviricetes	Stellavirales	Astroviridae	Mamastrovirus	Bat_astrovirus_Ha/Guangxi/LS11/2007
STT200	Rhinolophus shameli	2539	710	4.993893	ACN88714.1	58.6	239	277.3	Viruses	Riboviria	Orthornavirae	Pisuviricota	Stelpaviricetes	Stellavirales	Astroviridae	Mamastrovirus	Bat_astrovirus_Ha/Guangxi/LS11/2007
STT200	Rhinolophus shameli	8042	315	2.546154	ACN88713.1	86.9	99	195.3	Viruses	Riboviria	Orthornavirae	Pisuviricota	Stelpaviricetes	Stellavirales	Astroviridae	Mamastrovirus	Bat_astrovirus_Ha/Guangxi/LS11/2007
STT200	Rhinolophus shameli	6369	371	2.322785	ACN88714.1	86.1	108	184.9	Viruses	Riboviria	Orthornavirae	Pisuviricota	Stelpaviricetes	Stellavirales	Astroviridae	Mamastrovirus	Bat_astrovirus_Ha/Guangxi/LS11/2007
STT200	Rhinolophus shameli	13788	224	3.745562	ACN88713.1	79	62	104	Viruses	Riboviria	Orthornavirae	Pisuviricota	Stelpaviricetes	Stellavirales	Astroviridae	Mamastrovirus	Bat_astrovirus_Ha/Guangxi/LS11/2007
STT200	Rhinolophus shameli	1027	1401	3.322437	ACX83590.2	43.9	171	149.4	Viruses	Riboviria	Orthornavirae	Pisuviricota	Stelpaviricetes	Stellavirales	Astroviridae	Mamastrovirus	HMO_Astrovirus_A
STT200	Rhinolophus shameli	581	2179	20.885593	AAA42545.1	96.3	484	959.1	Viruses	Riboviria	Pararnavirae	Artverviricota	Revtraviricetes	Ortervirales	Retroviridae		AKT8_retrovirus
STT200	Rhinolophus shameli	6553	363	3.792208	ADI24415.1	98.3	120	240.7	Viruses	Riboviria	Pararnavirae	Artverviricota	Revtraviricetes	Ortervirales	Retroviridae		LNras*SN_acutely_transforming_retrovirus
STT200	Rhinolophus shameli	14677	216	2.503106	CAA25238.1	100	37	82	Viruses	Riboviria	Pararnavirae	Artverviricota	Revtraviricetes	Ortervirales	Retroviridae	Alpharetrovirus	Avian_carcinoma_Mill_Hill_virus_2
STT200	Rhinolophus shameli	4886	447	27.201531	P23091.1	77.5	102	145.2	Viruses	Riboviria	Pararnavirae	Artverviricota	Revtraviricetes	Ortervirales	Retroviridae	Alpharetrovirus	Avian_musculoaponeurotic_fibrosarcoma_virus_AS42
STT200	Rhinolophus shameli	8390	306	4.115538	P00527.2	96	101	213.8	Viruses	Riboviria	Pararnavirae	Artverviricota	Revtraviricetes	Ortervirales	Retroviridae	Alpharetrovirus	Y73_sarcoma_virus
STT200	Rhinolophus shameli	9862	276	6.289593	AAA43046.2	93.3	89	171.8	Viruses	Riboviria	Pararnavirae	Artverviricota	Revtraviricetes	Ortervirales	Retroviridae	Gammaretrovirus	Feline_leukemia_virus
STT200	Rhinolophus shameli	9789	277	10.31982	P01117.1	95.6	91	176	Viruses	Riboviria	Pararnavirae	Artverviricota	Revtraviricetes	Ortervirales	Retroviridae	Gammaretrovirus	Kirsten_murine_sarcoma_virus
STT200	Rhinolophus shameli	15452	209	6.701299	CAA80675.1	100	65	137.5	Viruses	Riboviria	Pararnavirae	Artverviricota	Revtraviricetes	Ortervirales	Retroviridae	Gammaretrovirus	Kirsten_murine_sarcoma_virus

Note 1. Bacteriophage hits not represented here.

Note 2.Best hits for each scaffold are chosen based on the highest percentage of amino acid sequence similarity.

Supplementary table 3. b. Viral scaffolds identified in this study for sample STT182.

sample_id	host_species	scaffold_id	scaffold_length_nt	scaffold_kmer_coverage	hit_accession	hit_pident_aa	hit_length_aa	hit_bitscore	hit_superkingdom	hit_realm	hit_kingdom	hit_phylum	hit_class	hit_order	hit_family	hit_genus	hit_species
STT182	Rhinolophus shameli	2426	1439	41.427023	ASV45858.1	64.1	382	511.9	Viruses								Angico_virus
STT182	Rhinolophus shameli	3436	1142	14.070837	AWY11113.1	34.3	175	100.9	Viruses								Chaq_virus
STT182	Rhinolophus shameli	8220	543	9.647541	ASA47393.1	34.9	169	100.1	Viruses								Culex_luteo-like_virus
STT182	Rhinolophus shameli	1979	1614	888.374599	ASA47518.1	32.6	172	87.4	Viruses								Culex_luteo-like_virus
STT182	Rhinolophus shameli	14581	316	7.762452	ASA47393.1	37.7	77	63.9	Viruses								Culex_luteo-like_virus
STT182	Rhinolophus shameli	7170	622	7.96649	P16836.1	91.8	61	126.3	Viruses	Duplodnaviria	Heunggongvirae	Peploviricota	Herviviricetes	Herpesvirales	Herpesviridae	Cytomegalovirus	Human_betaherpesvirus_5
STT182	Rhinolophus shameli	19278	253	3.479798	YP_009333370.1	54.9	71	92	Viruses	Riboviria							Beihai_barnacle_virus_12
STT182	Rhinolophus shameli	2462	1427	31.319971	YP_009329870.1	24.9	313	65.1	Viruses	Riboviria							Beihai_barnacle_virus_13
STT182	Rhinolophus shameli	14603	316	5.409962	YP_009330023.1	45	109	90.5	Viruses	Riboviria							Beihai_picorna-like_virus_73
STT182	Rhinolophus shameli	2709	1343	7.65295	YP_009337911.1	47.5	432	391.7	Viruses	Riboviria							Hubei_diptera_virus_13
STT182	Rhinolophus shameli	4124	995	8.168085	QJB76103.1	32.2	227	139	Viruses	Riboviria							Hubei_lepidoptera_virus_3
STT182	Rhinolophus shameli	10057	448	3.379135	YP_009330264.1	30.8	146	88.6	Viruses	Riboviria							Hubei_lepidoptera_virus_3
STT182	Rhinolophus shameli	21068	236	6.596685	APG78227.1	64.1	78	101.7	Viruses	Riboviria							Hubei_partiti-like_virus_10
STT182	Rhinolophus shameli	2106	1563	32.862069	APG78310.1	57.3	459	548.1	Viruses	Riboviria							Hubei_partiti-like_virus_40
STT182	Rhinolophus shameli	2726	1339	659.418224	APG78243.1	62.5	443	566.6	Viruses	Riboviria							Hubei_partiti-like_virus_46
STT182	Rhinolophus shameli	9161	491	3.337156	APG78218.1	53.7	164	173.7	Viruses	Riboviria							Hubei_partiti-like_virus_48
STT182	Rhinolophus shameli	2040	1586	25.834095	YP_009337365.1	37.6	186	112.1	Viruses	Riboviria							Hubei_permutotetra-like_virus_7
STT182	Rhinolophus shameli	173	4884	75.726859	YP_009337778.1	50.2	1125	1070.5	Viruses	Riboviria							Hubei_permutotetra-like_virus_9
STT182	Rhinolophus shameli	4971	860	5.269565	YP_009330068.1	42.8	250	201.4	Viruses	Riboviria							Hubei_sobemo-like_virus_33
STT182	Rhinolophus shameli	16900	280	1.613333	QKW94206.1	40.7	91	69.7	Viruses	Riboviria							PNG_bee_virus_4
STT182	Rhinolophus shameli	11902	381	4.125767	QJI53482.1	50.8	118	122.1	Viruses	Riboviria							Riboviria_sp.
STT182	Rhinolophus shameli	6865	650	6.796639	QJI53489.1	42.1	159	119.4	Viruses	Riboviria							Riboviria_sp.
STT182	Rhinolophus shameli	6350	696	5.034321	QKN89024.1	34	141	87.8	Viruses	Riboviria							Riboviria_sp.
STT182	Rhinolophus shameli	22875	222	3.347305	QDH90891.1	63.2	68	83.6	Viruses	Riboviria							Riboviria_sp.
STT182	Rhinolophus shameli	17051	278	2.103139	QKN88918.1	48.8	84	80.5	Viruses	Riboviria							Riboviria_sp.
STT182	Rhinolophus shameli	22302	226	4.502924	YP_009337329.1	50	62	57.8	Viruses	Riboviria							Sanxia_tombus-like_virus_6
STT182	Rhinolophus shameli	2088	1568	4.659617	YP_009337438.1	63.2	522	685.6	Viruses	Riboviria							Sanxia_water_strider_virus_9
STT182	Rhinolophus shameli	3082	1235	5.223729	YP_009337438.1	51.2	248	263.8	Viruses	Riboviria							Sanxia_water_strider_virus_9
STT182	Rhinolophus shameli	8266	541	3.399177	YP_009337438.1	60.6	180	225.7	Viruses	Riboviria							Sanxia_water_strider_virus_9
STT182	Rhinolophus shameli	13020	350	4.979661	YP_009337438.1	69	100	146	Viruses	Riboviria							Sanxia_water_strider_virus_9
STT182	Rhinolophus shameli	654	2709	29.189902	YP_009337273.1	50.2	297	295	Viruses	Riboviria							Shuangao_arthropod_virus_9
STT182	Rhinolophus shameli	2669	1355	530.236923	AVK59491.1	23	304	57.4	Viruses	Riboviria							Wuhan_fly_virus_6
STT182	Rhinolophus shameli	4881	871	9.884804	YP_009342451.1	43.3	261	187.6	Viruses	Riboviria							Wuhan_house_centipede_virus_4
STT182	Rhinolophus shameli	15901	294	3.845188	YP_009342462.1	46.4	69	63.5	Viruses	Riboviria							Wuhan_house_centipede_virus_4
STT182	Rhinolophus shameli	14342	321	3.834586	YP_009342464.1	46.3	108	95.1	Viruses	Riboviria							Wuhan_house_centipede_virus_5
STT182	Rhinolophus shameli	18557	260	6.043902	NP_149148.1	57.6	85	98.2	Viruses	Riboviria	Orthornavirae	Duplornaviricota	Resentoviricetes	Reovirales	Reoviridae	Cypovirus	Cypovirus_1
STT182	Rhinolophus shameli	10356	435	4.031579	AHJ14781.1	50.3	145	149.8	Viruses	Riboviria	Orthornavirae	Duplornaviricota	Resentoviricetes	Reovirales	Reoviridae	Cypovirus	Cypovirus_5
STT182	Rhinolophus shameli	13689	334	3.057348	AHJ14784.1	53.3	107	124	Viruses	Riboviria	Orthornavirae	Duplornaviricota	Resentoviricetes	Reovirales	Reoviridae	Cypovirus	Cypovirus_5
STT182	Rhinolophus shameli	13259	344	3.256055	AHJ14787.1	47.1	104	108.2	Viruses	Riboviria	Orthornavirae	Duplornaviricota	Resentoviricetes	Reovirales	Reoviridae	Cypovirus	Cypovirus_5
STT182	Rhinolophus shameli	15504	300	3.106122	AHJ14781.1	43.9	98	100.9	Viruses	Riboviria	Orthornavirae	Duplornaviricota	Resentoviricetes	Reovirales	Reoviridae	Cypovirus	Cypovirus_5
STT182	Rhinolophus shameli	10663	423	3.497283	AHJ14781.1	35.8	123	74.7	Viruses	Riboviria	Orthornavirae	Duplornaviricota	Resentoviricetes	Reovirales	Reoviridae	Cypovirus	Cypovirus_5
STT182	Rhinolophus shameli	17897	267	7.349057	YP_001883320.1	50	70	69.7	Viruses	Riboviria	Orthornavirae	Duplornaviricota	Resentoviricetes	Reovirales	Reoviridae	Cypovirus	Cypovirus_5
STT182	Rhinolophus shameli	18229	264	2.449761	YP_001883315.1	43.2	88	69.7	Viruses	Riboviria	Orthornavirae	Duplornaviricota	Resentoviricetes	Reovirales	Reoviridae	Cypovirus	Cypovirus_5
STT182	Rhinolophus shameli	14517	318	3.030418	AJC97791.1	42.9	105	105.1	Viruses	Riboviria	Orthornavirae	Duplornaviricota	Resentoviricetes	Reovirales	Reoviridae	Cypovirus	Thaumetopoea_pityocampa_cypovirus_5
STT182	Rhinolophus shameli	22412	225	5.676471	AJC97788.1	46.6	73	72.4	Viruses	Riboviria	Orthornavirae	Duplornaviricota	Resentoviricetes	Reovirales	Reoviridae	Cypovirus	Thaumetopoea_pityocampa_cypovirus_5
STT182	Rhinolophus shameli	4469	935	52.244318	AAM77034.1	95.1	142	263.1	Viruses	Riboviria	Orthornavirae	Kitrinoviricota	Flasuviricetes	Amarillovirales	Flaviviridae	Pestivirus	Pestivirus_A
STT182	Rhinolophus shameli	9102	493	23.360731	AAF73908.1	100	79	152.9	Viruses	Riboviria	Orthornavirae	Kitrinoviricota	Flasuviricetes	Amarillovirales	Flaviviridae	Pestivirus	Pestivirus_A
STT182	Rhinolophus shameli	16592	283	1857.346491	AAD44046.1	100	94	187.6	Viruses	Riboviria	Orthornavirae	Kitrinoviricota	Flasuviricetes	Amarillovirales	Flaviviridae	Pestivirus	Pestivirus_B
STT182	Rhinolophus shameli	1136	2150	26.383771	AXA52546.1	38.2	728	444.9	Viruses	Riboviria	Orthornavirae	Lenarviricota	Amabiliviricetes	Wolframvirales	Narnaviridae		Linepithema_humile_narna-like_virus_1
STT182	Rhinolophus shameli	12912	352	11.272727	AYP71797.1	71.6	109	171.8	Viruses	Riboviria	Orthornavirae	Lenarviricota	Miaviricetes	Ourlivirales	Botourmiaviridae	Ourmiavirus	Penicillium_citrinum_ourmia-like_virus_1
STT182	Rhinolophus shameli	13766	332	6.148014	AXA52551.1	67.3	110	164.5	Viruses	Riboviria	Orthornavirae	Pisuviricota	Duplopiviricetes	Durnavirales	Partitiviridae		Linepithema_humile_partiti-like_virus_1
STT182	Rhinolophus shameli	22559	224	4.402367	QDH88343.1	80.6	72	129.8	Viruses	Riboviria	Orthornavirae	Pisuviricota	Duplopiviricetes	Durnavirales	Partitiviridae		Partitiviridae_sp.
STT182	Rhinolophus shameli	18510	261	3.203883	AZT88595.1	56.5	85	105.5	Viruses	Riboviria	Orthornavirae	Pisuviricota	Duplopiviricetes	Durnavirales	Partitiviridae		Penicillium_digitatum_partitivirus_1
STT182	Rhinolophus shameli	2502	1413	116.303387	QIJ70089.1	54	437	484.6	Viruses	Riboviria	Orthornavirae	Pisuviricota	Duplopiviricetes	Durnavirales	Partitiviridae		Pennypacker_partiti-like_virus
STT182	Rhinolophus shameli	25011	208	1.431373	AWV67005.1	76.1	67	112.5	Viruses	Riboviria	Orthornavirae	Pisuviricota	Duplopiviricetes	Durnavirales	Picobirnaviridae		Lysoka_partiti-like_virus
STT182	Rhinolophus shameli	22073	228	3.023121	AWV67005.1	53.3	75	86.3	Viruses	Riboviria	Orthornavirae	Pisuviricota	Duplopiviricetes	Durnavirales	Picobirnaviridae		Lysoka_partiti-like_virus
STT182	Rhinolophus shameli	81	29798	101.709747	QIZ14016.1	95.6	4405	8315.3	Viruses	Riboviria	Orthornavirae	Pisuviricota	Pisoniviricetes	Nidovirales	Coronaviridae	Betacoronavirus	Severe_acute_respiratory_syndrome-related_coronavirus
STT182	Rhinolophus shameli	13558	337	3.088652	QKK82954.1	59	100	128.6	Viruses	Riboviria	Orthornavirae	Pisuviricota	Pisoniviricetes	Picornavirales			Erigeron_annuus_picorna-like_virus
STT182	Rhinolophus shameli	22194	227	3.226744	QNT09305.1	44.4	81	71.6	Viruses	Riboviria	Orthornavirae	Pisuviricota	Pisoniviricetes	Picornavirales			Hovenia_dulcis-associated_virus_2
STT182	Rhinolophus shameli	6225	708	4.894334	QKN88968.1	63	127	186.4	Viruses	Riboviria	Orthornavirae	Pisuviricota	Pisoniviricetes	Picornavirales			Picornavirales_sp.
STT182	Rhinolophus shameli	13790	332	2.981949	QKN88968.1	73.6	110	181.8	Viruses	Riboviria	Orthornavirae	Pisuviricota	Pisoniviricetes	Picornavirales			Picornavirales_sp.
STT182	Rhinolophus shameli	9571	471	3.086538	QKN88966.1	56.1	148	172.2	Viruses	Riboviria	Orthornavirae	Pisuviricota	Pisoniviricetes	Picornavirales			Picornavirales_sp.
STT182	Rhinolophus shameli	13894	329	11.047445	QKN88968.1	66.7	69	104.4	Viruses	Riboviria	Orthornavirae	Pisuviricota	Pisoniviricetes	Picornavirales			Picornavirales_sp.
STT182	Rhinolophus shameli	1901	1651	7.299499	AWK02674.1	58.9	518	598.6	Viruses	Riboviria	Orthornavirae	Pisuviricota	Pisoniviricetes	Picornavirales			Rhinolophus_picornavirus
STT182	Rhinolophus shameli	11547	392	1.967359	QKK82984.1	51.3	115	125.6	Viruses	Riboviria	Orthornavirae	Pisuviricota	Pisoniviricetes	Picornavirales			Teucrium_fruticans_picorna-like_virus
STT182	Rhinolophus shameli	15694	297	5.03719	AZB50981.1	40.5	74	71.2	Viruses	Riboviria	Orthornavirae	Pisuviricota	Pisoniviricetes	Picornavirales	Dicistroviridae		Bemisia-associated_dicistrovirus_1
STT182	Rhinolophus shameli	8316	538	6.291925	QJI52027.1	30.3	165	56.6	Viruses	Riboviria	Orthornavirae	Pisuviricota	Pisoniviricetes	Picornavirales	Dicistroviridae		Dicistroviridae_sp.
STT182	Rhinolophus shameli	13480	338	7.70318	QBS55240.1	40.2	82	71.6	Viruses	Riboviria	Orthornavirae	Pisuviricota	Pisoniviricetes	Picornavirales	Dicistroviridae		Tetranychus_urticae-associated_dicistrovirus_1
STT182	Rhinolophus shameli	117	8733	18.21664	NP_620555.1	33.1	1400	723.4	Viruses	Riboviria	Orthornavirae	Pisuviricota	Pisoniviricetes	Picornavirales	Dicistroviridae	Triatovirus	Plautia_stali_intestine_virus
STT182	Rhinolophus shameli	3799	1062	8.180735	AEM23662.1	75.7	354	575.1	Viruses	Riboviria	Orthornavirae	Pisuviricota	Pisoniviricetes	Picornavirales	Picornaviridae		Bat_picornavirus_3
STT182	Rhinolophus shameli	24082	213	10.411392	AET06141.1	52.9	70	72	Viruses	Riboviria	Orthornavirae	Pisuviricota	Pisoniviricetes	Picornavirales	Picornaviridae		Feline_picornavirus
STT182	Rhinolophus shameli	20891	238	4.562842	AFK85013.1	44	75	75.9	Viruses	Riboviria	Orthornavirae	Pisuviricota	Pisoniviricetes	Picornavirales	Picornaviridae		Rhinolophus_affinis_picornavirus_1
STT182	Rhinolophus shameli	5187	829	8.928941	AMX81409.1	57.5	275	327	Viruses	Riboviria	Orthornavirae	Pisuviricota	Pisoniviricetes	Picornavirales	Picornaviridae	Mischivirus	Mischivirus_D
STT182	Rhinolophus shameli	1436	1894	10.863513	AIF74258.1	67.6	238	342.4	Viruses	Riboviria	Orthornavirae	Pisuviricota	Pisoniviricetes	Picornavirales	Picornaviridae	Shanbavirus	Shanbavirus_A
STT182	Rhinolophus shameli	6583	673	5.883495	YP_009508298.1	60.8	222	265.8	Viruses	Riboviria	Orthornavirae	Pisuviricota	Pisoniviricetes	Picornavirales	Picornaviridae	Shanbavirus	Shanbavirus_A
STT182	Rhinolophus shameli	9696	465	2.202439	YP_009508298.1	42.4	151	132.1	Viruses	Riboviria	Orthornavirae	Pisuviricota	Pisoniviricetes	Picornavirales	Picornaviridae	Shanbavirus	Shanbavirus_A
STT182	Rhinolophus shameli	24287	212	6.318471	YP_009508298.1	52.9	70	77.8	Viruses	Riboviria	Orthornavirae	Pisuviricota	Pisoniviricetes	Picornavirales	Picornaviridae	Shanbavirus	Shanbavirus_A
STT182	Rhinolophus shameli	109	11328	19.728998	AXA52568.1	37.4	2252	1378.2	Viruses	Riboviria	Orthornavirae	Pisuviricota	Pisoniviricetes	Picornavirales	Polycipiviridae		Linepithema_humile_polycipivirus_1
STT182	Rhinolophus shameli	22035	228	4.606936	YP_009407937.1	38.5	78	62.4	Viruses	Riboviria	Orthornavirae	Pisuviricota	Pisoniviricetes	Picornavirales	Polycipiviridae	Sopolycivirus	Solenopsis_invicta_virus_4
STT182	Rhinolophus shameli	2854	1299	8.136656	QKI29238.1	32.5	203	88.6	Viruses	Riboviria	Orthornavirae	Pisuviricota	Pisoniviricetes	Sobelivirales	Solemoviridae		Amygdalus_persica_sobemo-like_virus
STT182	Rhinolophus shameli	634	2746	1290.837978	QOI91429.1	40.8	409	304.7	Viruses	Riboviria	Orthornavirae	Pisuviricota	Pisoniviricetes	Sobelivirales	Solemoviridae		Guangzhou_sobemo-like_virus
STT182	Rhinolophus shameli	7775	575	3.648077	QJI53804.1	44.4	180	146.4	Viruses	Riboviria	Orthornavirae	Pisuviricota	Pisoniviricetes	Sobelivirales	Solemoviridae		Solemoviridae_sp.
STT182	Rhinolophus shameli	18415	262	2.062802	QJI53807.1	60.9	69	84.3	Viruses	Riboviria	Orthornavirae	Pisuviricota	Pisoniviricetes	Sobelivirales	Solemoviridae		Solemoviridae_sp.
STT182	Rhinolophus shameli	843	2431	115.109007	AAA42545.1	96.3	484	959.1	Viruses	Riboviria	Pararnavirae	Artverviricota	Revtraviricetes	Ortervirales	Retroviridae		AKT8_retrovirus
STT182	Rhinolophus shameli	8518	525	8.140426	P00535.1	97.7	174	358.2	Viruses	Riboviria	Pararnavirae	Artverviricota	Revtraviricetes	Ortervirales	Retroviridae	Alpharetrovirus	Avian_leukosis_virus
STT182	Rhinolophus shameli	29021	99	3.704545	P00527.2	100	32	67	Viruses	Riboviria	Pararnavirae	Artverviricota	Revtraviricetes	Ortervirales	Retroviridae	Alpharetrovirus	Y73_sarcoma_virus
STT182	Rhinolophus shameli	7299	611	5.221223	AAA43042.1	99.5	203	414.1	Viruses	Riboviria	Pararnavirae	Artverviricota	Revtraviricetes	Ortervirales	Retroviridae	Gammaretrovirus	Feline_leukemia_virus
STT182	Rhinolophus shameli	12175	372	24.343849	YP_009507788.1	94.6	56	117.9	Viruses	Riboviria	Pararnavirae	Artverviricota	Revtraviricetes	Ortervirales	Retroviridae	Gammaretrovirus	Harvey_murine_sarcoma_virus
STT182	Rhinolophus shameli	1414	1908	13.366433	P01117.1	96.3	189	372.1	Viruses	Riboviria	Pararnavirae	Artverviricota	Revtraviricetes	Ortervirales	Retroviridae	Gammaretrovirus	Kirsten_murine_sarcoma_virus
STT182	Rhinolophus shameli	25133	207	2.934211	QDH76130.1	93.8	65	135.2	Viruses	Riboviria	Pararnavirae	Artverviricota	Revtraviricetes	Ortervirales	Retroviridae	Gammaretrovirus	Porcine_type-C_oncovirus
STT182	Rhinolophus shameli	19912	247	3.697917	QDH76130.1	80	80	109.8	Viruses	Riboviria	Pararnavirae	Artverviricota	Revtraviricetes	Ortervirales	Retroviridae	Gammaretrovirus	Porcine_type-C_oncovirus
STT182	Rhinolophus shameli	7883	567	3.736328	AVD69111.1	44.4	189	167.9	Viruses	Riboviria							HVAC-associated_RNA_virus_1
STT182	Rhinolophus shameli	11400	397	3.80117	AVD69111.1	59.4	133	157.5	Viruses	Riboviria							HVAC-associated_RNA_virus_1
STT182	Rhinolophus shameli	16571	284	2.812227	AVD69111.1	47.1	87	91.3	Viruses	Riboviria							HVAC-associated_RNA_virus_1
STT182	Rhinolophus shameli	17742	269	5.401869	AVD69111.1	36.8	87	64.7	Viruses	Riboviria							HVAC-associated_RNA_virus_1

Note 1. Bacteriophage hits not represented here.

Note 2.Best hits for each scaffold are chosen based on the highest percentage of amino acid sequence similarity.

Supplementary table 4. Primers used to confirm the sequence of the spike gene by SANGER sequencing.

Oligonucleotide Name	Oligonucleotide sequence (5' - 3')
SP-21490F	GGTAGACTCATCATTAGAGAC
SP-22410R	CCATTCTCATCAAAGCTGAGC
SP-22217F	GGTTTCTCTGTTCTACGACCA
SP-23213R	CACCTGTACCAGTTAAACCA
SP-22936F	GTCTGTTCTTAAGCCTTTTGA
SP-24002R	TGCTTGGTTTTGATGGATCTGGT
SP-23855F	CGTGCTTTAACTGGAATAGC
SP-24739R	ACCATGAGGTGCTGACTGAGG
SP-24617F	AGAGCTTCTGCTAATCTTGCT
SP-25527R	CCATCCGAAAGGGAGTGAGGCT

Supplementary table 5. List of the sequences used for phylogenetic studies.

Strain	Accession number (Genbank / GISAID)	Country	District	Host	Year
USA/CA1/2020	MN994467	USA	California	Human	2020
Wuhan-Hu-1/2019	MN908947	China	Wuhan	Human	2019
RacCS203	MW251308	Thailand	Chachoengsao	*Rhinolophus acuminatus*	2020
RpYN06	MZ081381	China	Yunnan	*Rhinolophus pusillus*	2020
bat_SL_CoVZC45	MG772933	China	Zhejiang - Zhoushan	*Rhinolophus pusillus*	2017
bat_SL_CoVZXC21	MG772934	China	Zhejiang - Zhoushan	*Rhinolophus pusillus*	2015
JTMC15	KU182964	China	Jilin	*Rhinolophus ferrumequinum*	2013
Rs3367	KC881006	China	Yunnan	*Rhinolophus sinicus*	2012
Shaanxi2011	JX993987	China	Shaanxi	*Rhinolophus pusillus*	2011
Yunnan2011	JX993988	China	Yunnan	*Chaerephon plicata*	2011
Longquan140	KF294457	China	Zhejiang	*Rhinolophus monoceros*	2012
GX2013	KJ473815	China	Guangxi	*Rhinolophus sinicus*	2013
Rs4237	KY417147	China	Yunnan	*Rhinolophus sinicus*	2013
Rs672	FJ588686	China	Guizhou	*Rhinolophus sinicus*	2006
SX2013	KJ473813	China	Shanxi	*Rhinolophus ferrumequinum*	2013
YN2013	KJ473816	China	Yunnan	*Rhinolophus sinicus*	2013
YNLF_31C	KP886808	China	Yunnan	*Rhinolophus ferrumequinum*	2013
Rf1	DQ412042	China	Hubei	*Rhinolophus ferrumequinum*	2004
LYRa11	KF569996	China	Yunnan	*Rhinolophus affinis*	2011
Rm1	DQ412043	China	Hubei	*Rhinolophus macrotis*	2004
HKU3_1	DQ022305	China	Hong Kong	*Rhinolophus sinicus*	2005
Rc_o319	LC556375	Japan	Iwate	*Rhinolophus cornutus*	2013
BtKY72	KY352407	Kenya		*Rhinolophus sp*	2007
WHU	AY394850	China	Hubei	Human	2003
BJ01	AY278488	China		Human	2003
Urbani	AY278741	USA		Human	2003
Rsh-STT182	EPI_ISL_852604	Cambodia	Steung Treng	*Rhinolophus shameli*	2010
Rsh-STT200	EPI_ISL_852605	Cambodia	Steung Treng	*Rhinolophus shameli*	2010
bat/Yunnan/RaTG13/2013	EPI_ISL_402131				
bat/Yunnan/RmYN01/2019	EPI_ISL_412976				
bat/Yunnan/RmYN02/2019	EPI_ISL_412977				
pangolin/Guangdong/1/2019	EPI_ISL_410721				
pangolin/Guangdong/P2S/2019	EPI_ISL_410544				
pangolin/Guangxi/P1E/2017	EPI_ISL_410539				
pangolin/Guangxi/P2V/2017	EPI_ISL_410542				
pangolin/Guangxi/P3B/2017	EPI_ISL_410543				
pangolin/Guangxi/P4L/2017	EPI_ISL_410538				
pangolin/Guangxi/P5E/2017	EPI_ISL_410541				
pangolin/Guangxi/P5L/2017	EPI_ISL_410540				

Supplementary table 6. GISAID acknowledgments table.

Accession ID	Originating laboratory	Submitting laboratory	Authors
EPI_ISL_402131	Wuhan Institute of Virology, Chinese Academy of Sciences	Wuhan Institute of Virology, Chinese Academy of Sciences	Yan Zhu, Ping Yu, Bei Li, Ben Hu, Hao-Rui Si, Xing-Lou Yang, Peng Zhou, Zheng-Li Shi
EPI_ISL_412976<br>EPI_ISL_412977	Shandong First Medical University & Shandong Academy of Medical Sciences	Institute of Microbiology, Chinese Academy of Sciences	Weifeng Shi, Tao Hu, Hong Zhou, Juan Li, Xing Chen, Alice Catherine Hughes, Yuhai Bi
EPI_ISL_410721	South China Agricultural University	South China Agricultural University	Yongyi Shen, Lihua Xiao, Wu Chen
EPI_ISL_410544<br>EPI_ISL_410539<br>EPI_ISL_410542<br>EPI_ISL_410543<br>EPI_ISL_410538<br>EPI_ISL_410541<br>EPI_ISL_410540	Beijing Institute of Microbiology and Epidemiology	Beijing Institute of Microbiology and Epidemiology	Wu-Chun Cao, Tommy Tsan-Yuk Lam, Na Jia, Ya-Wei Zhang, Jia-Fu Jiang, Bao-Gui Jiang

1 Furey, N., Bates, P.J.J., Bumrungsri, S., Francis, C., Csorba, G., Walston, J. & Thong, V.D. *Rhinolophus shameli*, <https://www.iucnredlist.org/species/19566/21993823> (2020).
2 Thong, V. D., Thanh, H.T., Soisook, P. & Csorba, G. *Rhinolophus acuminatus*, <https://www.iucnredlist.org/species/19520/21974227> (2019).
3 Furey, N., Walston, J., Kingston, T. & Hutson, A.M. *Rhinolophus affinis*, <https://www.iucnredlist.org/species/19522/21982358> (2020).
4 Fukui, D. *Rhinolophus pusillus*, <https://www.iucnredlist.org/species/85707059/21994916> (2019).
5 Bates, P., Bumrungsri, S., Csorba, G. & Soisook, P. *Rhinolophus malayanus*, <https://www.iucnredlist.org/species/19551/21978424> (2019).
