# Supplementary material for: A novel SARS-CoV-2 related coronavirus in bats from Cambodia
Source: Nat Commun. 2021 Nov 9;12:6563. doi: 10.1038/s41467-021-26809-4 (PMC8578604; doi:10.1038/s41467-021-26809-4)
Supplement: Supplementary file 3 — Reporting Summary [file 41467_2021_26809_MOESM3_ESM.pdf]

nature research

Corresponding author(s): Etienne SIMON-LORIERE and Veasna DUONG

Last updated by author(s): Sep 28, 2021

Reporting Summary

Nature Research wishes to improve the reproducibility of the work that we publish. This form provides structure for consistency and transparency in reporting. For further information on Nature Research policies, see our Editorial Policies and the Editorial Policy Checklist.

Statistics

For all statistical analyses, confirm that the following items are present in the figure legend, table legend, main text, or Methods section.

n/a	Confirmed	
☐	☒	The exact sample size (*n*) for each experimental group/condition, given as a discrete number and unit of measurement
☒	☐	A statement on whether measurements were taken from distinct samples or whether the same sample was measured repeatedly
☒	☐	The statistical test(s) used AND whether they are one- or two-sided<br>*Only common tests should be described solely by name; describe more complex techniques in the Methods section.*
☒	☐	A description of all covariates tested
☒	☐	A description of any assumptions or corrections, such as tests of normality and adjustment for multiple comparisons
☐	☒	A full description of the statistical parameters including central tendency (e.g. means) or other basic estimates (e.g. regression coefficient) AND variation (e.g. standard deviation) or associated estimates of uncertainty (e.g. confidence intervals)
☒	☐	For null hypothesis testing, the test statistic (e.g. *F*, *t*, *r*) with confidence intervals, effect sizes, degrees of freedom and *P* value noted<br>*Give P values as exact values whenever suitable.*
☒	☐	For Bayesian analysis, information on the choice of priors and Markov chain Monte Carlo settings
☒	☐	For hierarchical and complex designs, identification of the appropriate level for tests and full reporting of outcomes
☒	☐	Estimates of effect sizes (e.g. Cohen's *d*, Pearson's *r*), indicating how they were calculated

Our web collection on statistics for biologists contains articles on many of the points above.

Software and code

Policy information about availability of computer code

Data collection	Reference genome sequence data were downloaded from GenBank, ViPR and GISAID using the web interface.
Data analysis	Software used: Trimmomatic v0.39, SPAdes v3.14, megahit v1.2.9, CLC Assembly Cell v5.1.0, Geneious prime v2020.1.2, Ivar v1, DIAMOND v2.0.4, MAFTT v.7.467, MEGA v7, SimPlot v3.51, RDP v5, IQ-TREE v2.0.6, MrBayes v3.2.7, SWISS-MODEL (web)

For manuscripts utilizing custom algorithms or software that are central to the research but not yet described in published literature, software must be made available to editors and reviewers. We strongly encourage code deposition in a community repository (e.g. GitHub). See the Nature Research guidelines for submitting code & software for further information.

Data

Policy information about availability of data

All manuscripts must include a data availability statement. This statement should provide the following information, where applicable:

- Accession codes, unique identifiers, or web links for publicly available datasets
- A list of figures that have associated raw data
- A description of any restrictions on data availability

Sequence data that support the findings of this study have been deposited in the European Nucleotide Archive (Samples ERS5578105 and ERS5578106). The consensus sequences of RshSTT182 and RshSTT200 are also available at the GISAID with accession numbers: EPI_ISL_852604 and EPI_ISL_852605. The sequence of Rhinolophus shameli ACE2 gene has been deposited under GenBank accession number MZ851782. Data was retrieved from GenBank (https://www.ncbi.nlm.nih.gov/genbank/), GISAID (https://www.gisaid.org/), IUCN Red List of Threatened Species (https://www.iucnredlist.org/), Global Biodiversity Information Facility (https://www.gbif.org/).

Field-specific reporting

Please select the one below that is the best fit for your research. If you are not sure, read the appropriate sections before making your selection.

- [x] Life sciences
- [ ] Behavioural & social sciences
- [ ] Ecological, evolutionary & environmental sciences

For a reference copy of the document with all sections, see nature.com/documents/nr-reporting-summary-flat.pdf

Life sciences study design

All studies must disclose on these points even when the disclosure is negative.

Sample size	We screened 430 archived samples from 6 bat families and 2 carnivoran mammal families, including 162 oral swabs and 268 rectal swabs. Sixteen out of 430 (3.72%) samples tested positive for CoV by pan-CoV hemi-nested PCR. Two samples further tested positive using a RT-qPCR targeting the RdRp gene of sarbecoviruses, and both were processed for metagenomic sequencing.
Data exclusions	No data were excluded.
Replication	Sequencing of the spike was performed independently at 2 locations (France and Cambodia).
Randomization	Two of the 16 samples further tested positive using a RT-qPCR targeting the RdRp gene of sarbecoviruses, and no randomization was performed as both positive samples were sequenced.
Blinding	We only sequenced samples that were positive using a RT-qPCR targeting the RdRp gene of sarbecoviruses, and this was done using a metagenomic approach (not needing prior knowledge on the pathogen).

Reporting for specific materials, systems and methods

We require information from authors about some types of materials, experimental systems and methods used in many studies. Here, indicate whether each material, system or method listed is relevant to your study. If you are not sure if a list item applies to your research, read the appropriate section before selecting a response.

Materials & experimental systems

n/a	Involved in the study	
x		Antibodies
	x	Eukaryotic cell lines
x		Palaeontology and archaeology
	x	Animals and other organisms
x		Human research participants
x		Clinical data
x		Dual use research of concern

Methods

n/a	Involved in the study	
x		ChIP-seq
x		Flow cytometry
x		MRI-based neuroimaging

Eukaryotic cell lines

Policy information about cell lines

Cell line source(s)	HEK293T cells were purchased from Sigma.
Authentication	The purchase was recent, and we did not perform authentication of the cell line.
Mycoplasma contamination	The HEK293T cells tested negative for mycoplasma using the Venor GeM Advance kit.
Commonly misidentified lines (See ICLAC register)	No commonly misidentified cell lines were used in the study.

Animals and other organisms

Policy information about studies involving animals; ARRIVE guidelines recommended for reporting animal research

Laboratory animals	No laboratory animals were involved in the study.
Wild animals	Testing was performed on archived samples from several programs and field missions. In 2010, the Muséum national d'Histoire naturelle (MNHN, Paris, France) was mandated by UNESCO and the National Authority of Preah Vihear to conduct a mammal survey in northern Cambodia. During this mission, bats were captured using mist nets and harp traps in two provinces, Preah Vihear and Ratanakiri, to compare bat diversity on the two sides of the Mekong River. Oral and rectal swabs were collected

	from captured bats.<br>More recent sampling efforts were supported by the USAID-funded PREDICT projects which aimed to strengthen global capacity for detection and discovery of viruses with pandemic potential that can move between animals and people. In missions from 2012 to 2018, samples from bats and carnivorans were collected from free-ranging animals, private animal collection, restaurant, or hunted animals in Battambang, Kampong Cham, Mondulkiri, Preah Vihear, Pursat, Ratanakiri and Stung Treng. Mist nets were used to catch bats. Oral and rectal swabs were collected from live animals which were released after sampling.<br>The samples from the two sampling missions were stored in viral transport medium solution (VTM; containing tryptose phosphate Broth 2.95%, 145 mM of NaCl, 5% gelatin, 54 mM Amphotericin B, 106 U of penicillin-streptomycin per liter, 80 mg of gentamycin per liter [Sigma-Aldrich, Irvine, UK]) and were held in liquid nitrogen in dewars for transport to the Institut Pasteur du Cambodge where they were stored at −80 °C prior to testing.
Field-collected samples	Testing was performed on archived samples from several programs and field missions, all kept in freezers at -80°C for several years.
Ethics oversight	The study was approved by the General Directorate of Animal Health and Production and Forest Administration department of the Ministry of Agriculture Forestry and Fisheries in Cambodia. Sampling was conducted under a University of California, Davis Institutional Animal Care and Use Committee approved protocol (UC Davis IACUC Protocol No. 19300). The bat capture and sampling in 2010 was authorized by UNESCO and the National Authority of Preah Vihear.

Note that full information on the approval of the study protocol must also be provided in the manuscript.
